# Is laparoscope surgery feasible for upper gastrointestinal cancer patients with a history of abdominal surgery?

**DOI:** 10.3389/fsurg.2023.1214175

**Published:** 2023-10-09

**Authors:** Feng Li, Fan Zhang, Weixin Liu, Qingfeng Zheng, Moyan Zhang, Zhen Wang, Xuefeng Zhang, Ling Qi, Yong Li

**Affiliations:** ^1^ Department of Cardiothoracic Surgery, Neijiang Hospital of Traditional Chinese Medicine, Neijiang, China; ^2^ Department of Thoracic Surgery, National Cancer Center/National Clinical Research Center for Cancer/Cancer Hospital, Peking Union Medical College, Chinese Academy of Medical Sciences, Beijing, China; ^3^ Department of Thoracic Surgery, National Cancer Center/National Clinical Research Center for Cancer/Cancer Hospital Hebei Hospital, Chinese Academy of Medical Sciences, Langfang, China; ^4^ Department of Oncology, National Cancer Center/National Clinical Research Center for Cancer/Cancer Hospital, Peking Union Medical College, Chinese Academy of Medical Sciences, Beijing, China

**Keywords:** cancer of the esophagus, cancer of the gastroesophageal junction, history of abdominal surgery, laparoscopic secondary surgery, complications

## Abstract

**Objective:**

To investigate the feasibility of laparoscopic abdominal mobilization in patients with cancers of the esophagus or gastroesophageal junction who have a history of abdominal surgery.

**Methods:**

A total of 132 patients who underwent resection for cancers of the esophagus or gastroesophageal junction from August 2018 to March 2022 in the Department of Thoracic Surgery, Cancer Hospital, Chinese Academy of Medical Sciences, were selected (66 patients with a history of abdominal surgery (observation group) and 66 patients without a history of abdominal surgery (control group)). All patients were treated with preoperative neoadjuvant therapy, based on the clinical stage. Thoracoscopic and laparoscopic resection was performed under general anesthesia. The intraoperative and postoperative conditions and surgical complications were compared between the two groups.

**Results:**

No significant differences were found in baseline data between the observation group and the control group (*p *> 0.05). Laparoscopic abdominal mobilization was completed in both groups, and there were no significant differences between the two groups in the total operation time [(272.50 ± 86.45) min vs. (257.55 ± 67.96) min], abdominal mobilization time [(25.03 ± 9.82) min vs. (22.53 ± 3.88) min], blood loss [(119.09 ± 72.17) ml vs. (104.39 ± 43.82) ml], and postoperative time to first flatus [(3.44 ± 0.73) d vs. (3.29 ± 0.60) d] (*p *> 0.05). The abdominal mobilization time was longer in observation group than that in control group (*p *= 0.057). After excluding the patients (31/66) with a history of simple appendectomy from the observation group, the abdominal mobilization time was significantly longer in observation group than that in control group [(27.97 ± 12.16) min vs. (22.53 ± 3.88) min] (*p *< 0.05). There were significantly fewer dissected abdominal lymph nodes in the observation group than in the control group [(18.44 ± 10.87) vs. (23.09 ± 10.95), *p *< 0.05]. After excluding the patients (15/66) with a history of abdominal tumor surgery from the observation group, there was no significant difference in the number of dissected abdominal lymph nodes between the two groups [(20.62 ± 10.81) vs. (23.09 ± 10.95)] (*p *> 0.05).In addition, no postoperative complications, such as intestinal obstruction, abdominal infection and bleeding, occurred in either group.

**Conclusion:**

Patients with cancers of the esophagus or gastroesophageal junction who have a history of abdominal surgery are suitable for minimally invasive laparoscopic mobilization.

## 1. Introduction

Cancers of the esophagus and gastroesophageal junction are common upper gastrointestinal malignancies. In 2016, the morbidity and mortality rates of cancer of the esophagus were 8.2/100,000 and 12.37/100,000 in China, ranking sixth and fifth among malignancies, respectively (1). Surgery is the main treatment means for cancers of the esophagus and gastroesophageal junction. In particular, thoracoscopic and laparoscopic surgery has been widely used in clinical treatment because it is minimally invasive, with a rapid postoperative recovery, a short length of stay, and few pulmonary complications (2, 3). With the rising incidence of cancer, there have been increasingly more patients with secondary cancer or a history of abdominal surgery. Studies have shown that intestinal adhesions occur in approximately 90% of patients following abdominal surgery (4) and that isolating the adhesion produces a risk of injury to the intestinal tract, making surgical operations complex and secondary tumor resection difficult. At present, minimally invasive laparoscopic surgery has advantages with regard to the treatment of lower gastrointestinal tumor patients with a history of abdominal surgery. However, the effect of laparoscopic abdominal mobilization in patients with cancers of the esophagus or gastroesophageal junction who have a history of abdominal surgery remains unclear. In this study, 66 patients with cancers of the esophagus or gastroesophageal junction who had abdominal surgery from August 2018 to March 2022 were selected, and they underwent thoracoscopic and laparoscopic surgery and laparoscopic abdominal mobilization.

## 2. Materials and methods

### 2.1. General data

Patients in the Esophageal Ward of the Cancer Hospital, Chinese Academy of Medical Sciences, from August 2018 to March 2022, with resectable cancers of the esophagus or gastroesophageal junction and a history of abdominal surgery were selected as the observation group. Patients with a history of abdominal surgery at least once were enrolled, and those with a history of inguinal hernia repair, simple laparoscopic exploration, endoscopic therapy or planned open surgery were excluded. Finally, a total of 66 patients were included. Additionally, 66 patients during the same period with resectable cancers of the esophagus or gastroesophageal junction who had no history of abdominal surgery were randomly selected as the control group. The general data of patients in the observation group and control group are shown in Table 1.

**Table 1:** General data of the observation group and control group.

	Observation group (*n* = 66)	Control group (*n* = 66)	*p*
Sex (male)	57 (86.4)	55 (83.3)	0.627
Age (Y)	63.70 ± 9.46	61.12 ± 9.57	0.122
Body mass index (BMI)	22.94 ± 4.99	23.51 ± 4.80	0.509
Tumor type [*n* (%)]			0.307
Cancer of the esophagus	44 (66.7)	45 (68.2)	
Cancer of the gastroesophageal junction	19 (28.8)	21 (31.8)	
Postoperative recurrence	3 (4.5)	0 (0)	
Pathological T stage [*n* (%)]^a^			0.989
Tis	1 (1.5)	2 (3.0)	
T0	5 (7.6)	4 (6.1)	
T1	17 (25.8)	16 (24.2)	
T2	10 (15.2)	10 (15.2)	
T3	25 (37.9)	29 (43.9)	
T4	5 (7.6)	5 (7.6)	
Pathological N stage [*n* (%)]^a^			0.569
N0	33 (50.0)	28 (42.4)	
N1	15 (22.7)	16 (24.3)	
N2	7 (10.6)	8 (12.1)	
N3	8 (12.1)	14 (21.2)	
Neoadjuvant therapy [*n* (%)]	40 (60.6)	51 (77.3)	0.112
Neoadjuvant chemotherapy	24 (36.4)	36 (54.5)	
Neoadjuvant chemoradiotherapy^b^	4 (6.1)	3 (4.5)	
Neoadjuvant chemotherapy + immunotherapy	11 (16.7)	10 (15.2)	
Other neoadjuvant therapies	1 (1.5)	2 (3.0)	
ASA classification [*n* (%)]			0.778
I	0 (0)	1 (1.5)	
Ⅱ	58 (87.9)	59 (89.4)	
Ⅲ	8(12.1)	6(9.1)	
Ⅳ	0(0)	0(0)	

^a^ The patients in the observation group did not include 3 patients with postoperative recurrence.

^b^ The radiotherapy plan was not completed by one patient due to discontinuation of radiotherapy.

### 2.2. Methods

**Figure 1:**
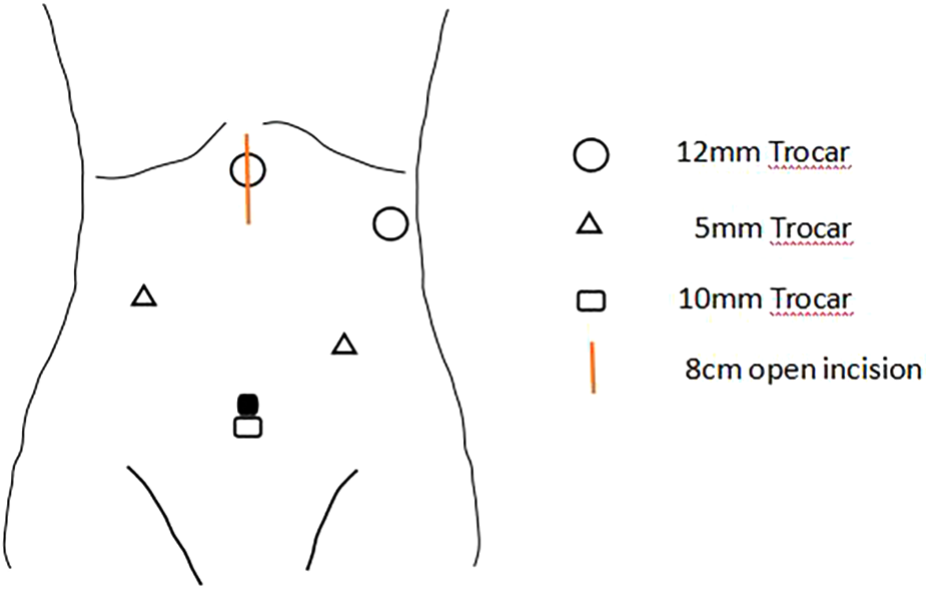
A 10 mm trocar will be placed at the navel for observation, and two 12 mm trocars will be placed at 2 cm below the rib edge on the left side and 2 cm below the xiphoid process, respectively. Two 5 mm Trocars will also be placed at the midpoint between the observation port and the Trocar on the left rib edge, and 6 cm below the right clavicle midline. After the stomach is freed, the incision below the xiphoid process will be extended to 8 cm, and the incision will be opened for gastric tube creation. The chief surgeon stands on the left side of the patient.

All patients were treated with neoadjuvant therapies, including chemoradiotherapy, chemotherapy, chemoradiotherapy + immunotherapy or chemotherapy + immunotherapy, based on the clinical stage. Thoracoscopic and laparoscopic resection was performed under general anesthesia. Specifically, McKeown surgery was performed for most cases of cancer of the esophagus, and Ivor-Lewis surgery was performed for some cases of cancer of the lower esophagus and most cases of cancer of the gastroesophageal junction. During abdominal mobilization, the chief surgeon standing on the left side of the patient made five semicircular incisions from the umbilicus to the bilateral subcostal arch and a small median incision in the abdomen, laparoscopically dissociated the stomach, omentum and blood vessels, and dissected abdominal lymph nodes. Finally, the stomach was taken out through the small median incision, and a tubular stomach was prepared under direct vision *in vitro* (Figure 1). For patients who required esophageal replacement with jejunum or colon, abdominal adhesions were laparoscopically released as far as possible, and the upper abdominal median incision was extended. The blood supply of the intestinal tract was determined under direct vision, and the appropriate intestinal segment was selected.

During abdominal mobilization in the observation group, a small incision far from the original surgical incision was first made. The first puncture point was as far as possible away from the original incision, by more than 5 cm, and the laparoscope was placed after finger exploration confirmed no abdominal adhesions. The second puncture point facilitated the separation of abdominal adhesions and provided conditions for the treatment of primary lesions; the puncture holes could be switched to facilitate adhesion separation and operation. There incisions were made at the non-adhesion site under the laparoscope, from which dissociation was completed. The abdominal mobilization time was calculated from the entry of the first trocar into the abdomen to the end of stomach dissociation and treatment with a tubular stomach, or to the end of treatment of intraoperative complications caused by dissociation, or to the end of exposure of the vascular arch and selection of an appropriate intestinal segment for esophageal replacement with jejunum or colon. Abdominal mobilization time, blood loss, number of dissected abdominal lymph nodes and perioperative complications were compared between the two groups.

### 2.3. Statistical analysis

SPSS 25.0 software was used for data analysis. Normally distributed data are presented as $\left(\bar{\chi }\pm s\right)$ and were compared using Student’s *t*-test. Non-normally distributed data are presented as medians (ranges) and were compared using the Mann-Whitney *U* test. Categorical variables are presented as [n(%)] and were compared using the chi-square test or Fisher’s exact probability test. *p *≤ 0.05 was considered statistically significant.

## 3. Results

In the observation group, there were 57 males (86.4%) and 9 females (13.6%) with a median age of 65 (30–83) years. In control group, there were 55 males (83.3%) and 11 females (16.7%) with a median age of 62.5 (32–86) years. No significant differences were found in baseline data such as body mass index (BMI), tumor type, pathological stage, preoperative neoadjuvant therapy, and ASA classification between the two groups (*p *> 0.05) (Table 2).

**Table 2:** History and type of surgery in the observation group.

History of surgery [*n* (%)]	
Once	52 (78.8)
Twice or more	14 (21.2)
Type of surgery [*n* (%)]	
Appendectomy	31 (47.0)
Cholecystectomy	15 (22.7)
Subtotal gastrectomy	4 (6.1)
Resection of colorectal cancer	4 (6.1)
Resection of cecal tumor	2 (3.0)
Laparoscopic resection of gastric stromal tumors	1 (1.5)
Intestinal repair of a stab wound	1 (1.5)
Cholecystectomy + pancreatic debridement and drainage	1 (1.5)
Appendectomy + resection of colon cancer	1 (1.5)
Appendectomy + hysterectomy	1 (1.5)
Cholecystectomy + choledochojejunostomy	1 (1.5)
Subtotal gastrectomy + gastric ESD	1 (1.5)
Simultaneous enterolysis + appendectomy	1 (1.5)
Resection of rectal cancer + uterine prolapse resection	1 (1.5)
Left thoracotomy for cardiac cancer	1 (1.5)

In the observation group, 52 patients (78.8%) had a history of abdominal surgery only once, and the remaining 14 patients (21.2%) had a history of more than one abdominal surgery. Specifically, 31 patients (47.0%) had undergone simple appendectomy, 15 patients (22.7%) had undergone cholecystectomy, 8 patients (12.1%) had undergone colorectal surgery, 6 patients (9.1%) had undergone gastric surgery, and 6 patients (9.1%) had undergone other surgeries (Table 2).

Laparoscopic abdominal mobilization was completed in both groups, and there were no significant differences between the two groups in the total operation time [(272.50 ± 86.45) min vs. (257.55 ± 67.96) min], blood loss [(119.09 ± 72.17) ml vs. (104.39 ± 43.82) ml], and postoperative time to first flatus [(3.44 ± 0.73) d vs. (3.29 ± 0.60) d] (*p *> 0.05). The abdominal mobilization time was longer in observation group than that in control group [(25.03 ± 9.82) min vs. (22.53 ± 3.88) min], but the difference was not significant (*p *= 0.057). After excluding the patients (31/66) with a history of simple appendectomy from the observation group, the abdominal mobilization time was significantly longer in the observation group than in the control group [(27.97 ± 12.16) min vs. (22.53 ± 3.88) min] (*p *< 0.05). There were significantly fewer dissected abdominal lymph nodes in the observation group than in the control group [(18.44 ± 10.87) vs. (23.09 ± 10.95), *p *< 0.05]. After excluding the patients (15/66) with a history of abdominal tumor surgery from the observation group, there was no significant difference in the number of dissected abdominal lymph nodes between the two groups [(20.62 ± 10.81) vs. (23.09 ± 10.95)] (*p *> 0.05) (Table 3).

**Table 3:** Comparison between the observation group and control group.

Clinical data	Observation group (*n* = 66)	Control group (*n* = 66)	*p*
Operation time (min)	272.50 ± 86.45	257.55 ± 67.96	0.271
Abdominal dissociation time (min)	25.03 ± 9.82	22.53 ± 3.88	0.057
Intraoperative blood loss (ml)	119.09 ± 72.17	104.39 ± 43.82	0.160
Intraoperative blood transfusion [*n* (%)]	4 (6.06)	2 (3.03)	0.680
Number of dissected abdominal lymph nodes (*n*)	18.44 ± 10.87	23.09 ± 10.95	0.016
Postoperative time to first flatus (d)	3.44 ± 0.73	3.29 ± 0.60	0.194
Postoperative complications [*n* (%)]	7 (10.6)	8 (12.1)	0.784
Pleural effusion	4 (6.0)	2 (3.0)	
Pulmonary infection	2 (3.0)	4 (6.0)	
Anastomotic fistula	1 (1.5)	0 (0)	
Respiratory failure requiring intubation	0 (0)	1 (1.5)	
Incision infection	0 (0)	1 (1.5)	
Intestinal hernia	0 (0)	1 (1.5)	
Arrhythmia	1 (1.5)	0 (0)	
Secondary surgery [*n* (%)]	0 (0)	0 (0)	
Postoperative ICU admission [*n* (%)]	2 (3.0)	5 (7.6)	0.440
Mode [*n* (%)]			0.270
Ivor-Lewis surgery	27 (40.9)	24 (36.4)	
McKeown surgery	34 (51.5)	41 (62.1)	
Transabdominal anastomosis via the diaphragmatic hiatus	4 (6.0)	1 (1.5)	
Anastomotic mode [*n* (%)]			0.604
Manual anastomosis	1 (1.5)	1 (1.5)	
Instrumental anastomosis	64 (97.0)	65 (98.5)	
Esophageal exclusion	1 (1.5)	0 (0)	
Replacement organ [*n* (%)]			0.078
Stomach	59 (89.4)	65 (98.5)	
Colon	3 (4.6)	1 (1.5)	
Jejunum	4 (6.0)	0 (0)	
Anastomotic site [*n* (%)]			0.081
Left neck	30 (45.5)	41 (62.1)	
Thoracic cavity	30 (45.5)	24 (36.4)	
Mediastinum	5 (7.6)	1 (1.5)	
Intraoperative complications [*n* (%)]	2 (3.0)	0 (0)	
Intestinal injury	1 (1.5)		
Rupture of the right gastroepiploic vessel	1(1.5)		

No postoperative complications, such as intestinal obstruction, abdominal infection or bleeding, occurred in either group. In the observation group, one patient who suffered adhesive intestinal obstruction secondary to past open cholecystectomy underwent vascular anastomosis due to damage to the right gastroepiploic vessel during mobilization and recovered well postoperatively. One patient with a jejunal injury recovered smoothly after repair.

## 4. Discussion

**Figure 2:**
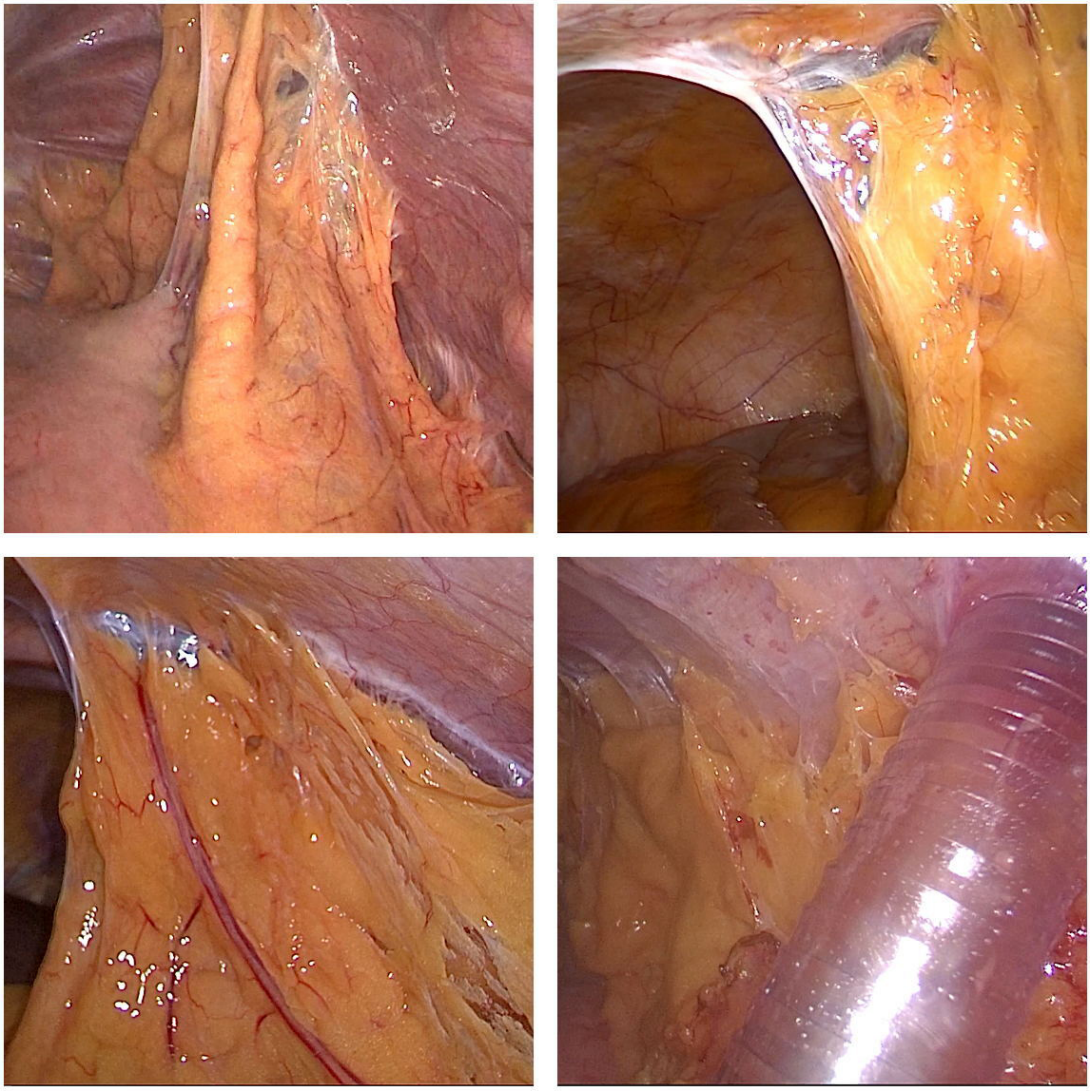
Various adhesions of the upper abdomen.

Abdominal adhesion is a common complication following abdominal surgery (Figure 2) and can lead to variations in the anatomical position of abdominal organs, reduce the surgical space due to insufficient pneumoperitoneum pressure, increase the difficulty of reoperation and the risk of intestinal or vascular injury during reoperation, and prolong the operation time (5). Therefore, a history of abdominal surgery has been listed as a relative contraindication to laparoscopic surgery (6). With the increase in life expectancy and tumors, however, patients with a history of abdominal surgery have become increasingly common, and surgeons have also been faced with challenges pertaining to reoperation.

With developments and improvements in technology and equipment as well as the continuous accumulation of operator experience, laparoscopic lysis of abdominal adhesions caused in previous abdominal surgery has become the first choice approach of increasingly more surgeons (7). The safety and effectiveness of thoracoscopic esophagectomy have been verified in previous studies (8, 9), and laparoscopic gastric mobilization has become a routine surgical procedure. Under magnification of the laparoscope system, the adjacent relationship of adhesions can be clearly exposed in the laparoscopic mobilization of abdominal adhesions, thus helping to avoid injury. However, there are few studies on laparoscopic abdominal mobilization and lymph node dissection in patients with cancers of the esophagus or gastroesophageal junction who have a history of abdominal surgery.

**Figure 3:**
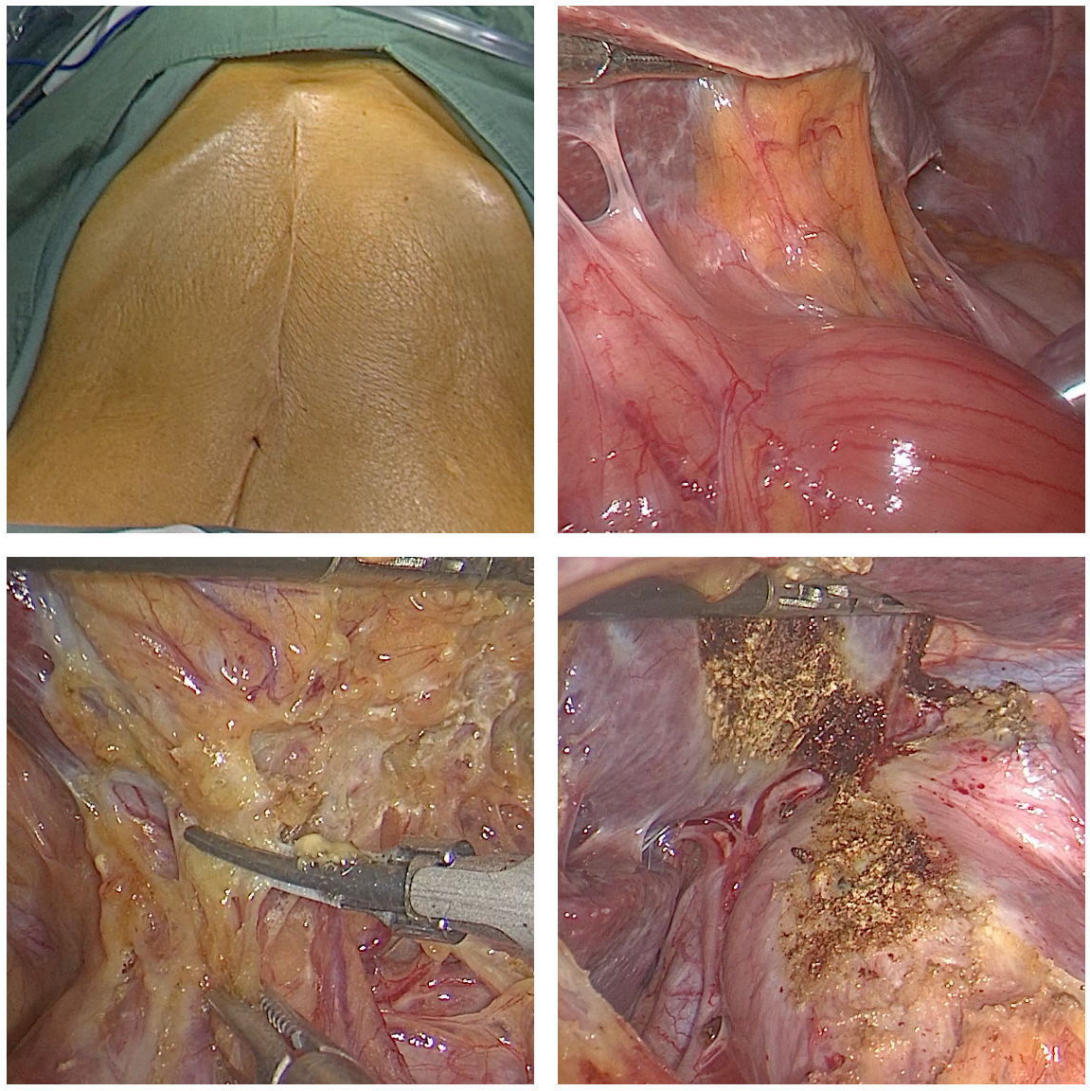
Reoperation for recurrence in patients with a history of transabdominal surgery for cardiac cancer. Operation time, 360 min; abdominal mobilization time, 35 min.

In this study, patients with cancers of the esophagus or gastroesophageal junction and a history of abdominal surgery underwent laparoscopic lysis of abdominal adhesions and stomach and abdominal lymph node dissection (Figure 3). The puncture point was selected to avoid injury to the abdominal viscera and blood vessels as much as possible. Additionally, a clear view under the laparoscope is required for the separation of adhesions. Adhesions were separated from intestinal vessels, and the spleen was protected. Ultrasonic scalpels were used for hemostasis. If the greater omentum was adhered to the anterior abdominal wall, especially when the pneumoperitoneum was perpendicular to the visual angle level, care was taken to avoid injury to the gastroepiploic vascular arch during mobilization. In the observation group, one patient underwent vascular repair and anastomosis due to damage to the right gastroepiploic vessel during mobilization and recovered well postoperatively. One patient with a jejunal injury recovered smoothly after repair. The two patients both had a history of upper abdominal surgery, illustrating again that abdominal adhesions caused by previous abdominal surgery increase the difficulty and risk of surgery. In particular, patients with a history of upper abdominal surgery should be closely monitored during abdominal adhesion lysis.

According to previous reports (8–13), thoracoscopic and laparoscopic esophagectomy can achieve the same effect as traditional open surgery, but it has obvious advantages such as less bleeding, mild postoperative pain, rapid recovery of gastrointestinal function and short length of stay. In this study, intraoperative blood loss was significantly lower in both groups than that previously reported for open surgery (14–16), but there was no significant difference in blood loss between the observation group and control group. In addition, postoperative recovery is faster for patients who undergo laparoscopic surgery than for those who undergo traditional open surgery, without increasing the incidence of postoperative complications (17–19). In this study, there were no significant differences between the observation group and control group in total operation time, abdominal mobilization time, blood loss, and postoperative time to first flatus, and neither surgical safety nor postoperative recovery were significantly different between the two groups. In this study, there were fewer abdominal lymph nodes in the observation group than in the control group. After excluding the patients (15/66) with a history of abdominal surgery from the observation group, there was no significant difference in the number of dissected abdominal lymph nodes between the two groups. A possible explanation for this result is that there were 13 patients (19.7%) with abdominal tumors in the observation group, and abdominal lymph node dissection during a previous surgery may affect the number of dissected abdominal lymph nodes in this study.

In this study, the mean operation time and abdominal mobilization time were longer in the observation group than in the control group, especially the latter, but there was no significant difference between the two groups. The possible reason is that the proportion of patients with a history of simple appendectomy was high (31/66) in the observation group. After excluding the patients with a history of simple appendectomy from the observation group, the abdominal mobilization time was significantly longer in the observation group than in the control group. As shown in previous studies, using McBurney’s point for laparoscopy or appendectomy may reduce the potential impact of adhesions following abdominal surgery (20). In this study, the degree of abdominal adhesions after simple appendectomy was significantly lower than that after other abdominal surgeries. Therefore, for patients with a history of abdominal surgery who undergo secondary surgery, the degree and impact of abdominal adhesions should be carefully determined before surgery based on the type of previous surgery, and the procedures and precautions for surgery should be planned to avoid secondary injuries as much as possible.

Certainly, there are some limitations in our study. Firstly, this is a retrospective cohort study, which may introduce selection bias. Secondly, we did not conduct further stratified analysis on cases with a history of previous abdominal surgery. The surgical approach for previous abdominal surgery, such as laparoscopic surgery and open surgery, can have a significant impact on postoperative abdominal adhesions.

In conclusion, previous surgery is not a contraindication to minimally invasive laparoscopic surgery for patients with cancers of the esophagus or gastroesophageal junction. A detailed preoperative inquiry of the surgical history, reasonable surgical planning, and care during the operation to avoid injury are necessary for laparoscopic lysis of abdominal adhesions to be an appropriate choice.

## Data Availability

The raw data supporting the conclusions of this article will be made available by the authors, without undue reservation.
